# Device and surgical procedure-related infections in Canadian acute care hospitals, 2018–2022

**DOI:** 10.14745/ccdr.v50i06a03

**Published:** 2024-06-28

**Authors:** Canadian Nosocomial Infection Surveillance Program

**Affiliations:** 1 Centre for Communicable Diseases and Infection Control, Public Health Agency of Canada, Ottawa, ON

**Keywords:** hospital-associated infection, acute care, surveillance, antimicrobial resistance, device-associated infection, surgical procedure-related infection, surgical site infection, CLABSI, central line-associated bloodstream infection, hip and knee arthroplasty surgical site infection, cerebrospinal fluid shunt surgical site infection, paediatric cardiac surgical site infection, Canada

## Abstract

**Background:**

Healthcare-associated infections (HAIs) are a significant healthcare burden in Canada. National surveillance of HAIs at sentinel acute care hospitals is conducted by the Canadian Nosocomial Infection Surveillance Program.

**Objective:**

This article describes device and surgical procedure-related HAI epidemiology in Canada from 2018 to 2022.

**Methods:**

Data were collected from over 60 Canadian sentinel acute care hospitals between January 1, 2018, and December 31, 2022, for central line-associated bloodstream infections (CLABSIs), hip and knee surgical site infections (SSIs), cerebrospinal fluid shunt (CSF) SSIs and paediatric cardiac SSIs. Case counts, rates, patient and hospital characteristics, pathogen distributions and antimicrobial resistance data are presented.

**Results:**

Between 2018 and 2022, 2,258 device-related infections and 987 surgical procedure-related infections were reported. A significant rate increase was observed in adult mixed intensive care unit CLABSIs (1.07–1.93 infections per 1,000 line days, *p*=0.05) and a non-significant rate increase was observed in SSIs following knee arthroplasty (0.31–0.42 infections per 100 surgeries, *p*=0.45). A fluctuating rate trend was observed in CSF shunt SSIs over the time period and a significant rate decrease in paediatric cardiac SSIs was observed (68%, from 7.5–2.4 infections per 100 surgeries, *p*=0.01). The most commonly identified pathogens were coagulase-negative staphylococci (22.8%) among CLABSIs and *Staphylococcus aureus* (42%) among SSIs.

**Conclusion:**

Epidemiological and microbiological trends among selected device and surgical procedure-related HAIs are essential for benchmarking infection rates nationally and internationally, identifying any changes in infection rates or antimicrobial resistance patterns and helping inform hospital infection prevention and control and antimicrobial stewardship policies and programs.

## Introduction

Healthcare-associated infections (HAIs) contribute to excess patient morbidity and mortality, leading to increased healthcare costs, longer hospital stays and increased antimicrobial resistance ((1)). Healthcare-associated infections may occur during the use of invasive devices and following surgical procedures ((2)). More specifically, surgical procedure-related infections are among the most prevalent HAIs and are responsible for a longer hospitalization of approximately seven to 11 days ((3)). Device and surgical procedure-related infections are also associated with a high-cost burden, accounting for almost $50,000 per central line-associated bloodstream infections (CLABSIs) case and $28,000 per surgical site infection (SSI) case ((4)).

A 2017 point prevalence study in Canadian sentinel acute care hospitals found that device and surgical procedure-related infections accounted for 35.6% of all reported HAIs ((5)). Central line-associated bloodstream infections accounted for 21.2% of device and surgical procedure-related infections while prosthetic implants accounted for 19.4% ((5)). The risk of device and surgical procedure-related infections is associated with patient demographics and comorbidities, in addition to the type of hospital in which the patient received care ((6–8)).

Understanding the epidemiology of device and surgical procedure-related HAIs is essential to provide benchmark rates over time, which help to inform effective antimicrobial stewardship and infection prevention and control measures. In addition, the collection and analysis of antimicrobial susceptibility data are important to inform the appropriate use of antimicrobials and help reduce antimicrobial resistance ((9)). This report provides an epidemiological overview of select device and surgical procedure-related HAIs from 2018 to 2022 in over 60 hospitals participating in the Canadian Nosocomial Infection Surveillance Program (CNISP).

## Methods

### Design

Since its establishment in 1994, CNISP has conducted national HAI surveillance at sentinel acute care hospitals across Canada, in collaboration with the Public Health Agency of Canada and the Association of Medical Microbiology and Infectious Disease Canada (AMMI Canada). Data are presented for the following device and surgical procedure-related HAIs: CLABSIs; hip and knee arthroplasty SSIs; cerebrospinal fluid (CSF) shunt SSIs; and paediatric cardiac SSIs.

### Case definitions

Device and surgical procedure-related HAIs were defined according to standardized protocols and case definitions (see **Appendix**). Complex infections, defined as deep incisional and organ/space, were included in hip and knee SSI surveillance, while CLABSIs identified in intensive care unit (ICU) settings were included in CLABSI surveillance. The adult mixed ICU, adult cardiovascular surgery intensive care unit (CVICU), paediatric intensive care unit (PICU) and neonatal intensive care unit (NICU) were included as eligible ICU settings. Adult mixed intensive care units included any adult ICU with a mix of patient types as part of the ICU patient mix (i.e., medical/surgical, surgical/trauma, burn/trauma, medical/neurosurgical).

### Data source

Epidemiological data for device and surgical procedure-related infections identified between January 1, 2018, and December 31, 2022 (using surgery date for surgical site infections and date of positive blood culture for CLABSIs) were submitted by participating hospitals using standardized data collection forms. Hospital participation varied by surveillance project and year. Data submission and case identification were supported by training sessions and periodic evaluations of data quality.

### Statistical analysis

To calculate hip and knee SSI, CSF shunt SSI and paediatric cardiac SSI rates, the number of cases were divided by the number of surgical procedures performed (multiplied by 100). To calculate CLABSI rates, the number of cases was divided by line day denominators (multiplied by 1,000). Neonatal intensive care unit CLABSI rates stratified by birth weight category were not included in this report. To calculate proportions of pathogens, the number of pathogens were divided by the total number of identified pathogens. Denominators may vary, as missing and incomplete data were excluded from analyses. Median and interquartile ranges (IQR) were calculated for continuous variables. Trends over time were tested using the Mann-Kendall test. Significance testing was two-tailed and differences were considered significant at a *p*-value of ≤0.05. Analyses were conducted using R version 4.1.2 and SAS 9.4.

## Results

Over 60 hospitals contributed device and surgical procedure-related infection data to CNISP between 2018 and 2022 (**Table 1**), with medium-sized (n=201−499 beds) adult hospitals (n=16 sites, 25%) being the most common (data not shown). Overall, 2,258 device-related infections and 987 surgical procedure-related infections were reported. Among all SSIs reported (n=987), hip and knee infections represented 68% (n=667) of these types of infections.

**Table 1:** Characteristics of acute care hospitals participating in device and surgical procedure-related healthcare-associated infection surveillance, 2022

Characteristic of hospitals	CLABSI-adult mixed ICU	CLABSI-adult CVICU	CLABSI-PICU	CLABSI-NICU	CSF shunt SSI	Paediatric cardiac SSI	Hip and knee SSI	Total unique hospitals
Total number of participating hospitals	36	8	12	18	16	6	32	64
**Hospital type**								
Adult	27	6	N/A	4^a^	4	N/A	15	33
Mixed	9	2	4	6	2	N/A	17	22
Paediatric	N/A	N/A	8	8	10	6	N/A	9
**Hospital size**								
Small (1–200 beds)	2	1	7	8	8	3	6	18
Medium (201–499 beds)	22	3	4	7	5	3	15	31
Large (500 or more beds)	12	4	1	3	3	N/A	11	15

Abbreviations: CLABSI, central line-associated bloodstream infection; CSF shunt SSI, cerebrospinal fluid shunt surgical site infection; CVICU, cardiovascular surgery intensive care unit; ICU, intensive care unit; N/A, not applicable; NICU, neonatal intensive care unit; PICU, paediatric intensive care unit; SSI, surgical site infection

^a^ Four hospitals classified as “adult” also had a NICU

A total of 2,496 pathogens were identified from device-related infections and 1,056 pathogens from surgical procedure-related cases between 2018 and 2022. Of the identified pathogens for CLABSIs, 61% were gram-positive, 24% were gram-negative and 15% were fungal. Of the identified pathogens for SSIs, 79% were gram-positive, 19% were gram-negative and 1.5% were fungal. Coagulase-negative staphylococci (CoNS) and *Staphylococcus aureus* were the most frequently reported pathogens for CLABSIs and SSIs, respectively (**Table 2**). From 2018 to 2022, the proportion of methicillin-resistant *S. aureus* (MRSA) was 16% for CLABSIs and 11% for SSIs (data not shown).

**Table 2:** Distribution and rank of the five most frequently reported gram negative, gram-positive and fungal pathogens, 2018–2022^a^

Pathogen category	Rank	Pathogen	CLABSI N=2,258		Hip and knee N=667		CSF shunt N=151		Paediatric cardiac N=169	
			n	%	n	%	n	%	n	%
Gram-positive	1	Coagulase-negative staphylococci^b^	568	22.8	143	18.9	58	35.6	22	16.3
	2	*Staphylococcus aureus* ^c^	257	10.3	288	38.0	49	30.1	77	57.0
	3	*Enterococcus* spp.	536	21.5	33	4.4	6	3.7	1	0.7
	4	*Streptococcus* spp.	58	2.3	69	9.1	4	2.5	9	6.7
	Other gram-positive^d^		94	3.8	64	8.4	13	8.0	0	0.0
	Total gram-positive		1,513	60.6	597	78.8	130	79.8	109	80.7
Gram-negative	1	*Klebsiella* spp.	139	5.6	18	2.4	8	4.9	3	2.2
	2	*Escherichia coli*	126	5.0	26	3.4	8	4.9	1	0.7
	3	*Enterobacter* spp.	99	4.0	34	4.5	3	1.8	5	3.7
	4	*Pseudomonas* spp.	67	2.7	29	3.8	4	2.5	3	2.2
	5	*Serratia* spp.	46	1.8	11	1.5	2	1.2	2	1.5
	Other gram-negative^e^		133	5.3	40	5.3	5	3.1	2	1.5
	Total gram-negative		610	24.4	158	20.8	30	18.4	16	11.9
Fungi	1	*Candida albicans*	189	7.6	2	0.3	1	0.6	3	2.2
	2	Other *Candida* spp.^f^	175	7.0	1	0.1	2	1.2	6	4.4
	Other fungi^g^		9	0.4	0	0.0	0	0.0	1	0.7
	Total fungal		373	14.9	3	0.4	3	1.8	10	7.4
Total			2,496	N/A	758	N/A	163	N/A	135	N/A

Abbreviations: CLABSI, central line-associated bloodstream infections; CSF shunt, cerebrospinal fluid shunt; N/A, not applicable

^a^ Frequency distribution percentage rounded to the nearest tenth decimal

^b^ Coagulase-negative staphylococci included *S. lugdunensis*, *S. haemolyticus*, *S. epidermidis*, *S. capitis*, *S. hominis* and *S. warneri*

^c^ *Staphylococcus aureus* includes methicillin-resistant *S. aureus*, methicillin-susceptible *S. aureus* and unspecified *S. aureus*

^d^ Other gram-positive pathogens included anaerobic gram-positive cocci, *Finegoldia magna*, *Clostridioides* spp., *Lactobacillus* spp. and others

^e^ Other gram-negative pathogens included *Stenotrophomonas* spp., *Morganella morganii*, *Proteus mirabilis*, *Pantoea* spp., *Prevotella* spp., *Bacteroides fragilis* and others

^f^ Other *Candida* spp. included *C. dubliniensis*, *C. glabrata*, *C. krusei*, *C. lusitaniae*, *C. parapsilosis* and *C. tropicalis*

^g^ Other fungi included *Aspergillus* spp., *Trichophyton tonsurans* and unspecified fungi

### Central line-associated bloodstream infections

A total of 2,258 CLABSIs were reported between 2018 and 2022, with the majority occurring in adult mixed ICUs (n=1,411, 62.5%) and NICUs (n=456, 20.2%). Overall, NICUs had the highest rates of CLABSIs between 2018 and 2022 (1.75 infections per 1,000 line days), followed by adult mixed ICUs (1.66 infections per 1,000 line days), PICUs (1.65 infections per 1,000 line days) and adult CVICUs (0.82 infections per 1,000 line days) (**Table A1**).

**Figure 1:**
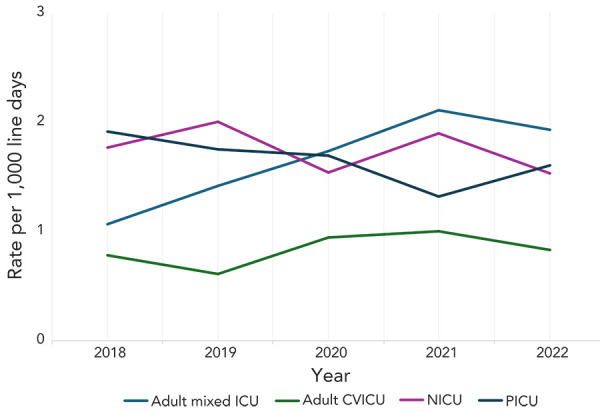
Rate of central line-associated bloodstream infection per 1,000 line days by intensive care unit type, 2018–2022 Abbreviations: CVICU, cardiovascular intensive care unit; ICU, intensive care unit; NICU, neonatal intensive care unit; PICU, paediatric intensive care unit

From 2018 to 2022, CLABSI rates fluctuated in NICUs and PICUs, while CLABSI rates in adult mixed ICUs increased significantly by 80% (1.07–1.93 infections per 1,000 line days, *p*=0.05) (**Figure 1**). Though rates of CLABSI in adult CVICUs were low overall, adult CVICU CLABSI rates increased 28% from 2018 to 2021 (0.78–1.0 infections per 1,000 line days) before decreasing 20% to 0.83 infections per 1,000 line days in 2022.

Among CLABSIs identified in adult mixed ICUs, the median age was 60 years (IQR=47–69 years), with males representing the majority of cases (66%). All-cause mortality within 30 days following the first positive culture, for adult mixed ICU CLABSI patients was 32% (n=452/1,411). Among CLABSIs identified in adult CVICUs, the median age was 65 years (IQR=51–72 years), with males representing 72% of cases. Within 30 days following the first positive culture, all-cause mortality for adult CVICU CLABSI patients was 29.1% (n=39/134). Among CLABSIs identified in PICUs, the median age was seven months (IQR=3−36 months), with males representing 58% of cases. Within 30 days following the first positive culture, all-cause mortality for PICU CLABSI patients was 8.9% (n=23/257). Among CLABSIs identified in NICUs, the median age at first positive culture was 19 days (IQR=9−41 days). Males represented 59% of NICU cases and all-cause mortality within 30 days of positive culture was 12% (n=53/456).

The most commonly identified pathogens among CLABSIs overall were CoNS and *Enterococcus* spp. (22.8% and 21.5%, respectively), which aligned with the most commonly identified pathogens among adult mixed ICUs and adult CVICUs. Among PICU and NICU CLABSIs, CoNS and *S. aureus* were the most commonly identified pathogens (data not shown). Among CLABSIs identified with *Serratia* spp., most were in the adult mixed ICU (54.3%, n=25/46), followed by NICU (17.4%, n=8/46), PICU (17.4%, n=8/46) and adult CVICUs (10.9%, n=5/46).

### Hip and knee surgical site infections

**Figure 2:**
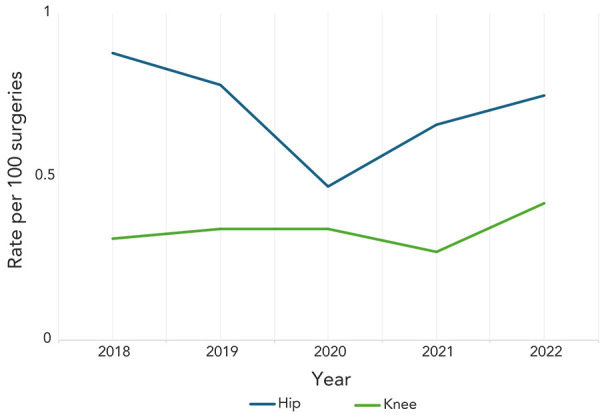
Rate of hip and knee surgical site infections per 100 surgeries, 2018–2022

A total of 667 complex hip and knee SSIs were reported between 2018 and 2022, of which the majority were hip arthroplasties (n=440, 66%). Among hip and knee SSIs, 55% (n=242) were organ/space infections and 45% (n=198) were deep incisional infections (**Table 3**). From 2018 to 2022, knee SSI rates increased non-significantly by 35.5% (0.31–0.42 infections per 100 surgeries, *p*=0.45) while hip SSI rates fluctuated between 0.75 and 0.88 infections per 100 surgeries (*p*=0.33) (**Figure 2**). During the COVID-19 pandemic in 2020, knee SSI rates remained stable while hip SSI rates decreased by 40%, compared to 2019. In 2022, both hip and knee SSI rates increased to 0.72 and 0.42 infections per 100 surgeries respectively, returning to rates observed in the pre-pandemic period (Figure 2 and **Table A2**).

**Table 3:** Frequency of hip and knee surgical site infections by year and infection type, 2018–2022

Year	Deep incisional SSI		Organ/space SSI		All cases
	n	%	n	%	n
**Hip arthroplasty**					
2018	34	34.7	64	65.3	98
2019	52	50.0	52	50.0	104
2020	22	44.9	27	55.1	49
2021	44	49.4	45	50.6	89
2022	46	46.0	54	54.0	100
Overall	198	45.0	242	55.0	440
**Knee arthroplasty**					
2018	22	55.0	18	45.0	40
2019	27	50.9	26	49.1	53
2020	14	37.8	23	62.2	37
2021	23	62.2	14	37.8	37
2022	33	55.0	27	45.0	60
Overall	119	52.4	108	47.6	227

Abbreviation: SSI, surgical site infection

The median patient age was 68 years (IQR=59–75 years) for hip SSIs and 66 years (IQR=59–74 years) for knee SSIs. The median time from procedure to hip and knee infections was 22 days (IQR=15–34 days) and 24 days (IQR=16–39 days), respectively. For data collected between 2018 and 2022, the median length of stay was three days (IQR=1–7 days) for hip SSIs and two days (IQR=1–4 days) for knee SSIs. Most patients (84%, n=552/661) with an SSI following hip or knee arthroplasty were readmitted and 66% (n=431/652) required revision surgery. Within 30 days after first positive culture, five all-cause deaths (2.1%, n=9/427) were reported among patients with a complex SSI following a hip arthroplasty while zero all-cause deaths were reported among patients with a knee arthroplasty SSI. Among hip and knee SSI cases, *S. aureus* and CoNS were the most commonly identified pathogens at 38% and 19%, respectively, and did not differ by deep or organ/space infection type (data not shown).

### Cerebrospinal fluid shunt surgical site infections

**Figure 3:**
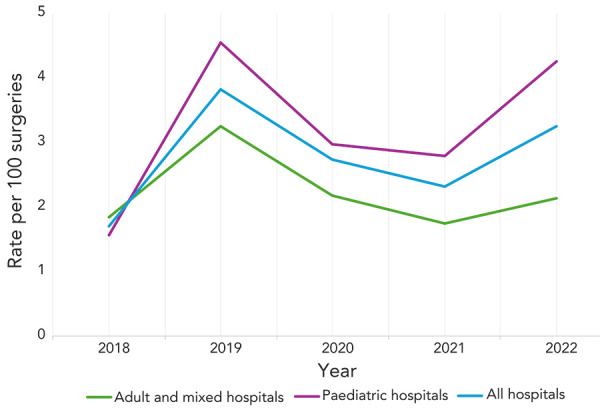
Cerebrospinal fluid shunt surgical site infection rates per 100 surgeries by hospital type^a^, 2018–2022 ^a^ All hospitals include adult, mixed and paediatric hospitals participating in cerebrospinal fluid shunt surgical site infection surveillance

Between 2018 and 2022, 151 CSF shunt SSIs were reported, with an overall rate of 2.9 infections per 100 surgeries (range: 1.7–3.82 infections per 100 surgeries, **Table A3**). Paediatric and adult/mixed hospitals infection rates were not significantly different at 3.2 and 2.5 infections per 100 surgeries, respectively (*p*=0.17). Cerebrospinal fluid shunt SSI rates in all hospitals decreased throughout the COVID-19 pandemic in 2020 and 2021 (**Figure 3**), then increased by 41% in 2022 (2.3 infections per 100 surgeries in 2021 to 3.3 infections per 100 surgeries in 2022). Paediatric hospital CSF shunt SSI rates decreased by 39% from 2019 to 2021, before increasing again to 4.3 infections per 100 surgeries in 2022, in keeping with the fluctuating rate trend observed since 2011 (data not shown).

More than half of CSF shunt SSIs (53.6%, n=81/151) were identified from new surgeries while 46.4% (n=70/151) were identified from revision surgeries. The median age was 47 years (IQR=36–62 years) for adult patients and three years (IQR=0.4–9 years) for paediatric patients. Females represented 54% (n=82/151) of cases and median time from surgery to infection was 19 days (IQR=10–40 days). The most commonly identified pathogens from CSF shunt SSIs were CoNS and *S. aureus* (36% and 30% of identified pathogens, respectively). Outcome data were not collected for CSF shunt SSI surveillance.

### Paediatric cardiac surgical site infections

**Figure 4:**
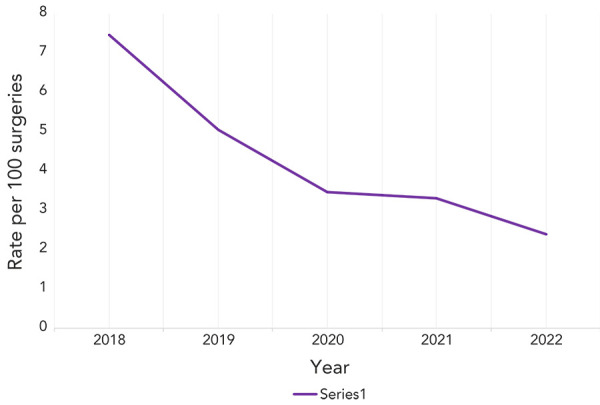
Paediatric cardiac surgical site infection rates per 100 surgeries, 2018–2022

A total of 169 paediatric cardiac SSIs were reported between 2018 and 2022 (**Table 4**). Most of these SSIs were superficial infections (62%), followed by organ/space infections (30%). Overall, the average paediatric cardiac SSI rate was 3.9 infections per 100 surgeries (**Table A4**). From 2018 to 2022, rates decreased significantly by 68% and consistently, from 7.5 to 2.4 infections per 100 surgeries (*p*=0.01) (**Figure 4**). The high rate in 2018 was caused by outlier cases attributable to two hospitals.

**Table 4:** Paediatric cardiac surgical site infection rates by year and infection type, 2018–2022

Year	Superficial incisional SSI cases		Organ/space SSI cases		Deep incisional SSI cases		All cases^a^
	n	%	n	%	n	%	
2018	18	46.2	15	38.5	6	15.4	39
2019	19	54.3	14	40.0	2	5.7	35
2020	29	78.4	6	16.2	2	5.4	37
2021	23	65.7	9	25.7	3	8.6	35
2022	15	65.2	6	26.1	2	8.7	23
Overall	104	61.5	50	29.6	15	8.9	169

Abbreviation: SSI, surgical site infection

^a^ Excludes cases with missing infection type information

The median age of patients with a paediatric cardiac SSI was 40 days (IQR=6–246 days) and the median time from surgery to onset date of infection was 16 days (IQR=8–24 days). Among the three deaths reported within 30 days of infection onset (1.8% of cases), one death was unrelated to the paediatric cardiac SSI, while two deaths were attributable to the paediatric cardiac SSI. *Staphylococcus aureus* and CoNS were the most commonly identified pathogens from paediatric cardiac SSIs (57% and 16% of identified pathogens, respectively) and did not differ by superficial, organ/space or deep infection type (data not shown).

### Antibiogram

**Figure 5:**
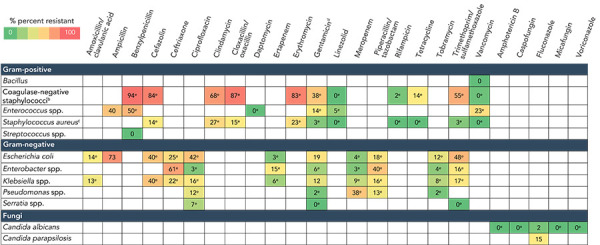
Antibiogram results^a^ from pathogens identified from central line-associated bloodstream infections, 2018–2022^b,c,d,e^ ^a^ Antibiotic/organism combinations with fewer than 30 tests were excluded ^b^ Coagulase-negative staphylococci included *S. lugdunensis, S. haemolyticus, S. epidermidis, S. capitis, S. hominis* and *S. warneri* ^c^ Included methicillin-susceptible *S. aureus* and methicillin-resistant *S. aureus* (MRSA) ^d^ Gentamicin synergy for gram-positive organisms ^e^ Less than 90% of isolates were tested

**Figure 6:**
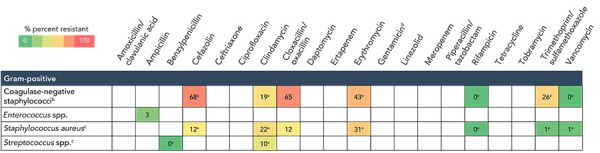
Antibiogram results^a^ from pathogens identified from hip and knee, cerebrospinal fluid shunt and paediatric cardiac surgical site infections, 2018–2022^b,c,d,e^ ^a^ Antibiotic/organism combinations with fewer than 30 tests were excluded ^b^ Coagulase-negative staphylococci included *S. lugdunensis, S. haemolyticus, S. epidermidis, S. capitis, S. hominis* and *S. warneri* ^c^ Included methicillin-susceptible *S. aureus* and methicillin-resistant *S. aureus* (MRSA) ^d^ Gentamicin synergy for gram-positive organisms ^e^ Less than 90% of isolates were tested

Results of antimicrobial susceptibility testing for the most frequently identified gram-positive, gram-negative and fungal pathogens from device and surgical procedure-related HAIs are listed in **Figure 5** and **Figure 6**. The *S. aureus* isolates were resistant to cloxacillin/oxacillin (MRSA) in 15% (n=28/189) of CLABSIs and 12% (n=40/337) of SSIs. Meropenem resistance ranged from 3% to 38% in gram-negative pathogens identified from CLABSIs. No meropenem resistance was observed among pathogens isolated from SSIs. Seventy-six vancomycin-resistant *Enterococci* were identified among CLABSIs (23%).

## Discussion

This report summarizes 2,258 device-related infections and 987 surgical procedure-related infections identified over five years of surveillance (2018–2022) from 64 hospitals across Canada. During this time, rates of device and surgical procedure-related HAIs have increased significantly by 80% for adult mixed ICU CLABSIs and non-significantly by 36% for knee SSIs. The COVID-19 pandemic has had a varied impact on the rates of device and surgical procedure-related HAIs ((10)). In Canada, preliminary investigations suggest that the COVID-19 pandemic had an immediate but unsustained impact on HAI rate trends ((11)). Rates of SSIs in the CNISP network initially decreased in 2020 during the COVID-19 pandemic, when elective surgeries were postponed, before increasing towards pre-pandemic levels in 2021. Ongoing investigations continue to assess the influence of pandemic-related factors such as changes in infection control practices, screening, laboratory testing and antimicrobial stewardship on the observed rates of HAIs.

### Central line-associated bloodstream infections

Where comparable data were available, the rates of CLABSI in adult ICUs (overall rate: 0.82 and 1.66 infections per 1,000 line days for CVICUs and mixed ICUs, respectively) were lower than those in the United Kingdom but higher than those in Western Australia ((12–14)). In the United Kingdom, 2021 and 2022 rates of CLABSI in the adult and cardiac ICU were 2.5 and 1.6 infections per 1,000 line days, respectively ((14)). In Western Australia, CLABSI rates in adult ICU settings ranged from 0.0 to 0.8 infections per 1,000 line days between 2018 and 2022 and may be lower than levels in Canada due to differences in surveillance methodologies including the number and type of hospitals under surveillance ((12)). Compared to CNISP adult mixed ICU CLABSI rates, a European Centre for Disease Prevention and Control report noted similar or higher 2019 rates in France and Italy (1.4–3.8 infections per 1,000 line days), while Austrian and Lithuanian CLABSI rates were lower (0.1–0.2 infections per 1,000 line days) ((15)).

Rates of CLABSIs in the NICU and PICU fluctuated from 2018 to 2022 but were higher overall (1.75 and 1.65 infections per 1,000 line days, respectively) compared to CLABSI rates in adult mixed ICUs and adult CVICUs (1.66 and 0.82 infections per 1,000 line days, respectively). Data available from the United States from 2018 to 2022 indicate the standardized incidence ratios (defined as the ratio of observed number of infections compared to the 2015 baseline) have reported similar fluctuating trends and have experienced a 9% decrease in CLABSI rates between 2021 and 2022 ((16–20)). Higher rates of CLABSIs have been seen in other limited resource settings compared to those observed in the CNISP network; a large surveillance study of ICUs in 45 countries from Latin America, Europe, Eastern Mediterranean, Southeast Asia and Western Pacific World Health Organization regions reported pooled mean CLABSI rates of 5.37 per 1,000 line days in PICUs (57 participating ICUs) and 4.66 in medical/surgical adult ICUs (182 participating ICUs) between January 2015 and December 2020 ((21)).

### Surgical site infections

Among SSIs included in this surveillance report, hip and knee SSIs were the most prevalent. Hip SSI rates fluctuated across reporting years, while knee SSI rates increased non-significantly. Surveillance from the United Kingdom indicates hip and knee SSI rates slightly increased for 2021 and 2022, after remaining stable for 10 years ((22)). Compared to CNISP data, hip and knee SSI rates reported in Southern Australia were higher overall and have also seen increases in recent years; hip SSI rates increased from 2018 to 2020 (1.80–1.91 infections per 100 procedures), while knee SSI rates increased from 0.79 to 0.88 infections per 100 procedures, during the same time period ((23)). In accordance with results from other regions, the most common pathogens among hip and knee SSIs were *S. aureus* and CoNS, likely attributed to the contamination of implant devices by the patient’s endogenous skin flora ((24,25)). Higher median age of patients with hip and knee SSIs relate to the older age of patients requiring joint replacements and the increased likelihood of surgical complications ((26)). Our data indicate that frequent readmission and revision surgeries are required for SSIs, both of which place high economic and resource burdens on the Canadian healthcare system, consistent with other studies from the United States, Australia and the United Kingdom ((27–30)).

The overall rate of SSIs from CSF shunts was 2.85 per 100 surgeries from 2018 to 2022. Stratification of CSF shunt SSI data by paediatric and adult/mixed hospitals showed that from 2018 to 2022, adult rates (2.5 infections per 100 surgeries) and paediatric rates (3.2 infections per 100 surgeries) were not significantly different. Data from historical CNISP surveillance shows a fluctuating trend in CSF shunt SSI rates from 2011 to 2020 ((31)). Compared to historical data, CSF shunt SSI rates among paediatric patients from 2018 to 2022 (3.2%) were lower than those from 2000 to 2002 (4.9%), signifying a decrease in SSI rates among paediatric populations ((32)). The rate of CSF shunt SSI among adult patients from 2018 to 2022 (2.5%) was also lower compared to that of 2000 to 2002 (3.2%) ((32)). A national survey from 2017 conducted in England showed a mean brain shunt infection rate of 1.9% (range: 0–4.4%), which is lower than what we observed, although there may be variations in the definitions and methodologies of rate calculation ((33)).

The overall rate of paediatric cardiac SSI between 2018 and 2022 was 3.93 per 100 surgeries. The relatively high rate of paediatric cardiac SSI in 2018 should be interpreted with caution, as rates may fluctuate due to the limited number of annual cases. Literature regarding paediatric cardiac SSI rates is limited; however, a pre-post intervention study from 2013 to 2017 has reported successful reduction in paediatric cardiac SSI rates from 3.4 to 0.9 per 100 surgeries in a quaternary, paediatric academic center in California following the implementation of a postoperative SSI reduction care bundle ((34)).

### Antibiogram

The percentage of *S. aureus* isolates that were MRSA among CLABSIs (15%) and SSIs (12%) was lower in the CNISP network compared to data reported by Centers for Disease Control and Prevention where 44% and 38% of *S. aureus* isolates were MRSA for CLABSIs and SSIs, respectively ((35)).

Of the identified *Enterococcus* spp. in CLABSIs, 23% were vancomycin-resistant *Enterococci* (VRE). From National Healthcare Safety Network surveillance in the United States, 73% of *Enterococcus faecium* and 4% of *Enterococcus faecalis* pathogens identified from CLABSIs in ICUs were VRE in 2021 ((36)). Meropenem resistance was low in most gram-negative pathogens identified among CLABSIs and SSIs (0%–8%) in the CNISP network, and similar to carbapenem resistance levels reported in the United States in 2021 (5% among *Klebsiella* spp.; 6% among *Enterobacter* spp.; and 0.8% among tested *E. coli* isolates) ((37)).

However, among *Pseudomonas* spp. identified in CLABSIs, meropenem resistance was 38%, which is higher than levels reported in the United States (21% carbapenem-resistant *Pseudomonas aeruginosa* among CLABSIs in 2021) ((38,39)). Overall, antibiogram patterns observed in the CNISP network may differ compared to other countries due to differences in surveillance methodologies, antimicrobial stewardship practices, types of hospitals or patient populations under surveillance and differences in circulating molecular strain types.

## Strengths and limitations

The main strength of CNISP surveillance is the standardized collection of detailed epidemiological and molecular linked data from a large representative network of sentinel hospitals across Canada. From 2018 to 2022, CNISP coverage of Canadian acute care beds has increased from 32% to 35%, including increased representativeness in northern, community, rural, and Indigenous populations. To further improve representativeness, CNISP has launched a simplified dataset accessible to all acute care hospitals across Canada to collect and visualize annual HAI rate data. The number of hospitals participating in each HAI surveillance project differed and epidemiologic data collected were limited to the information available in the patient charts. For CLABSI surveillance, data were limited to infections occurring in the ICU settings, and as such may only represent a subset of CLABSIs occurring in the hospital. Further, differences in surveillance protocols and case definitions limit comparison with data from other countries. Studies are ongoing to assess the impact of the COVID-19 pandemic on device and surgical procedure-related HAIs and antimicrobial resistance.

## Conclusion

This report provides an updated summary of rates, pathogen distributions and antimicrobial resistance patterns among select device and surgical procedure-related HAIs and relevant pathogens. The collection and analysis of national surveillance data are important to understanding and reducing the burden of device and surgical procedure-related HAIs. These data provide benchmark rates for national and international comparison and inform antimicrobial stewardship and infection prevention and control programs and policies.

## Appendix Case definitions

### Central line-associated bloodstream infection

Only central line-associated bloodstream infections (CLABSIs) related to an intensive care unit (ICU) admission were included in surveillance.

Bloodstream infections case definition:

Bloodstream infection is **NOT** related to an infection at another site and it meets one of the following criteria:

**Criterion 1:** Recognized pathogen cultured from at least one blood culture, unrelated to infection at another site.

OR

**Criterion 2:** At least one of: fever (higher than 38°C core), chills, hypotension; if aged younger than 1 year, fever (higher than 38°C core), hypothermia (lower than 36°C core), apnea or bradycardia **AND** common skin contaminant (see list below) cultured from at least two blood cultures drawn on separate occasions or at different sites, unrelated to infection at another site. Different sites may include peripheral veins, central venous catheters or separate lumens of a multilumen catheter. Different times include two blood cultures collected on the same or consecutive calendar days via separate venipunctures or catheter entries. The collection date of the first positive blood culture is the date used to identify the date of positive culture. Two positive blood culture bottles filled at the same venipuncture or catheter entry constitute only one positive blood culture.

Central line-associated bloodstream infection case definition:

A CLABSI must meet one of the following criteria:

**Criterion 1:** A laboratory-confirmed bloodstream infection (LCBSI) where a central line catheter (CL) or umbilical catheter (UC) was in place for more than two calendar days on the date of the positive blood culture, with day of device placement being Day 1.

OR

**Criterion 2:** A LCBSI where a CL or UC was in place more than two calendar days and then removed on the day or one day before positive blood culture was drawn.

Intensive care unit-related central line-associated bloodstream infection case definition:

A CLABSI related to an ICU if it meets one of the following criteria:

**Criterion 1:** CLABSI onset after two days of ICU stay.

OR

**Criterion 2:** If the patient is discharged or transferred out of the ICU, the CLABSI would be attributable to the ICU if it occurred on the day of transfer or the next calendar day after transfer out of the ICU.

Note: If the patient is transferred into the ICU with the CL and the blood culture was positive on the day of transfer or the next calendar day, then the CLABSI would be attributed to the unit where the line was inserted.

Common skin contaminants:

Diphtheroids, *Corynebacterium* spp., *Bacillus* spp., *Propionibacterium* spp., coagulase-negative staphylococci (including *S. epidermidis*), viridans group streptococci, *Aerococcus* spp., *Micrococcus* spp. and *Rhodococcus* spp.

### Hip and knee surgical site infection

Only complex surgical site infections (SSIs) (deep incisional or organ/space) following hip and knee arthroplasty were included in surveillance.

A deep incisional surgical site infection must meet the following criterion:

Infection occurs within 90 days after the operative procedure and the infection appears to be related to the operative procedure and involves deep soft tissues (e.g., facial and muscle layers) of the incision and the patient has at least **ONE** of the following:

· Purulent drainage from the deep incision but not from the organ/space component of the surgical site

· Deep incision that spontaneously dehisces or is deliberately opened by the surgeon and is culture-positive or not cultured when the patient has at least one of the following signs or symptoms: fever (higher than 38°C) or localized pain or tenderness (a culture-negative finding does not meet this criterion)

· An abscess or other evidence of infection involving the deep incision is found on direct examination, during reoperation or by histopathologic or radiologic examination

· Diagnosis of a deep incisional SSI by a surgeon or attending physician

An organ/space surgical site infection must meet the following criterion:

Infection occurs within 90 days after the operative procedure and the infection appears to be related to the operative procedure and infection involves any part of the body, excluding the skin incision, fascia or muscle layers, that is opened or manipulated during the operative procedure and patient has at least **ONE** of the following:

· Purulent drainage from a drain that is placed through a stab wound into the organ/space

· Organisms isolated from an aseptically obtained culture of fluid or tissue in the organ/space

· An abscess or other evidence of infection involving the organ/space that is found on direct examination, during reoperation or by histopathologic or radiologic examination

· Diagnosis of an organ/space SSI by a surgeon or attending physician

### Cerebrospinal fluid shunt surgical site infection

Only patients who underwent a placement or revision of a cerebrospinal fluid (CSF) shunting device and the infection occurred within one year of surgery were included in surveillance.

Cerebrospinal fluid shunt-associated surgical site infection case definition:

An internalized CSF shunting device is in place **AND** a bacterial or fungal pathogen(s) is identified from the cerebrospinal fluid **AND** is associated with at least **ONE** of the following:

· Fever (temperature 38°C or higher)

· Neurological signs or symptoms

· Abdominal signs or symptoms

· Signs or symptoms of shunt malfunction or obstruction

### Paediatric cardiac surgery surgical site infection

Only surgical site infections following open-heart surgery with cardiopulmonary bypass among paediatric patients (younger than 18 years of age) were included in surveillance.

A **superficial incisional SSI** must meet the following criterion: Infection occurs within 30 days after the operative procedure and involves only skin and subcutaneous tissue of the incision and meets at least **ONE** of the following criteria:

· Purulent drainage from the superficial incision

· Organisms isolated from an aseptically obtained culture of fluid or tissue from the superficial incision

· At least **ONE** of the following signs or symptoms of infection:

o Pain or tenderness, localized swelling, redness or heat, and the superficial incision is deliberately opened by a surgeon, and is culture-positive or not cultured (a culture-negative finding does not meet this criterion)

o Diagnosis of superficial incisional SSI by the surgeon or attending physician

A **deep incisional SSI** must meet the following criterion:

Infection occurs within 90 days after the operative procedure and the infection appears to be related to the operative procedure **AND** involves deep soft tissues (e.g., facial and muscle layers) of the incision **AND** the patient has at least **ONE** of the following:

· Purulent drainage from the deep incision but not from the organ/space component of the surgical site

· Deep incision spontaneously dehisces or is deliberately opened by the surgeon and is culture-positive or not cultured when the patient has at least one of the following signs or symptoms: fever (higher than 38°C) or localized pain or tenderness (a culture-negative finding does not meet this criterion)

· An abscess or other evidence of infection involving the deep incision is found on direct examination, during reoperation or by histopathologic or radiologic examination

· Diagnosis of a deep incisional SSI by a surgeon or attending physician

An **organ/space SSI** must meet the following criterion:

Infection occurs within 90 days after the operative procedure and the infection appears to be related to the operative procedure **AND** infection involves any part of the body, excluding the skin incision, fascia or muscle layers, that is opened or manipulated during the operative procedure **AND** the patient has at least **ONE** of the following:

· Purulent drainage from a drain that is placed through a stab wound into the organ/space

· Organisms isolated from an aseptically obtained culture of fluid or tissue in the organ/space

· An abscess or other evidence of infection involving the organ/space that is found on direct examination, during reoperation or by histopathologic or radiologic examination

**Table A1:** Rate of central line-associated bloodstream infection per 1,000 line days by intensive care unit type, 2018–2022

Year	Adult mixed ICU	Adult CVICU	NICU	PICU
2018	1.07	0.78	1.77	1.92
2019	1.42	0.61	2.01	1.75
2020	1.74	0.95	1.54	1.70
2021	2.11	1.00	1.90	1.32
2022	1.93	0.83	1.53	1.61
Overall	1.66	0.82	1.75	1.65

Abbreviations: CVICU, cardiovascular intensive care unit; ICU, intensive care unit; NICU, neonatal intensive care unit; PICU, paediatric intensive care unit

**Table A2:** Rate of hip and knee surgical site infections per 100 surgeries, 2018–2022

Year	Hip	Knee
2018	0.88	0.31
2019	0.78	0.34
2020	0.47	0.34
2021	0.66	0.27
2022	0.75	0.42
Overall	0.71	0.34

**Table A3:** Cerebrospinal fluid shunt surgical site infection rates per 100 surgeries by hospital type, 2018–2022

Year	Adult and mixed hospitals	Paediatric hospitals	All hospitals^a^
2018	1.84	1.56	1.70
2019	3.25	4.55	3.82
2020	2.17	2.97	2.73
2021	1.75	2.79	2.31
2022	2.14	4.26	3.25
Overall	2.50	3.15	2.85

^a^ All hospitals include adult, mixed, and paediatric hospitals participating in cerebrospinal fluid shunt surgical site infection surveillance

**Table A4:** Paediatric cardiac surgical site infection rates per 100 surgeries, 2018–2022

Year	Rate
2018	7.46
2019	5.04
2020	3.46
2021	3.31
2022	2.38
Overall	3.93
